# High Prevalence of Work-Related Musculoskeletal Disorders Among Dentists: A Cross-Sectional Study From Kerala, India

**DOI:** 10.7759/cureus.70254

**Published:** 2024-09-26

**Authors:** PM Gopika, Silpa T Sasi, Jeby J Olickal, Kavumpurathu R Thankappan

**Affiliations:** 1 Public Health, Amrita Institute of Medical Sciences, Kochi, IND; 2 Community Medicine, Amrita Institute of Medical Sciences, Kochi, IND

**Keywords:** dentists, ergonomic, musculoskeletal disorders, occupational health doctor, work ability

## Abstract

Background: Literature on the prevalence of musculoskeletal disorders (MSD), work ability, and associated factors among dentists is limited. Therefore, this study aimed to determine the prevalence of MSD, work ability, and associated factors among dentists in Kerala, India.

Methods: This cross-sectional study was conducted among 290 dentists (median age: 34 years, 47% male), selected from a list of dentists in a district of Kerala. Information on MSD and work ability were collected using a structured, pre-tested questionnaire administered both online and through direct interviews. Factors associated with MSD were analyzed using multivariable regression analysis.

Results: The prevalence of MSD in the past 12 months was 86.9% (95% CI: 83.1 - 90.9), and in the past seven days was 42.4% (95% CI: 36.7 - 48.08). Among those reporting MSD in the last 12 months, 21.4% experienced it in two sites. Of the nine regions assessed, the neck was the most affected over the past 12 months, with 57.6% reporting discomfort, while the lower back was the most affected in the last seven days (21.4%). Work ability was rated as excellent by 24.5% of participants and good by 51.7%. In the regression analysis, no significant association was found between socioeconomic factors and MSD during either time period. However, bivariate analysis showed that dentists who were overweight and those aged 34 years or older reported significantly more knee-related MSD in the past seven days (p=0.033 and p=0.037, respectively).

Conclusion: Despite the high prevalence of MSD among dentists, more than three-fourths reported good or excellent work ability. Implementing mandatory ergonomic assessments and interventions in dental workplaces may help reduce MSD among dentists.

## Introduction

Musculoskeletal disorders (MSD) involve injuries or conditions impacting muscles, nerves, tendons, joints, cartilage, and spinal discs [1]. According to the World Health Organization, these conditions are characterized by pain and mobility limitations, leading to decreased work capacity and social participation among affected individuals. Analysis of the Global Burden of Disease (GBD) 2019 data revealed that approximately 1.71 billion individuals worldwide are affected by musculoskeletal conditions [2]. These conditions include a range of issues, such as low back pain, neck pain, fractures, injuries, osteoarthritis, amputation, and rheumatoid arthritis [2]. The prevalence of MSD among dental professionals in Western countries ranged from 10.8% to 97.9% [3]. In India, the prevalence of work-related MSD (WMSD) among dentists ranges from 19.4% to 100% [4].

WMSD specifically refers to conditions where the work environment and job performance play a significant role in their development. These disorders may be exacerbated or prolonged due to specific conditions present in the workplace. Various work conditions, such as frequent lifting of heavy objects, regular exposure to whole-body vibration, repetitive overhead work, prolonged periods of neck flexion, and performing forceful tasks repeatedly [5], contribute to a high prevalence of WMSD among dentists. The profession demands outstanding visual clarity, auditory acuity, depth perception, psychomotor abilities, and manual dexterity. Additionally, dentists are required to maintain occupational postures for extended periods, which further increases their susceptibility to MSD. Consequently, dentists are more prone to such conditions compared to professionals in other fields [6]. Diminution of any of these abilities affects the practitioner’s performance and productivity. Even with significant progress in dentistry, various occupational health issues continue to endure in the field [7].

The concept of work ability encompasses the alignment between an individual's physical, mental, social, environmental, and organizational requirements of their job and their ability to effectively fulfill these demands. Work ability is subject to the influence of multiple factors, including the working environment, personal attributes, surrounding conditions, and socio-cultural aspects. While studies on MSD exist, there are limited or no studies specifically focusing on WMSD and work ability among dentists in Kerala. Therefore, we aimed to study the prevalence of MSD, work ability, and associated factors among dentists in Thrissur district, Kerala.

## Materials and methods

Study design and setting

This cross-sectional study was conducted among dentists affiliated with the Indian Dental Association (IDA) in Thrissur district, Kerala.

Study population and duration

The study included dentists registered with the IDA in Thrissur who had at least 12 months of work experience. Data collection occurred over seven months, from March 1, 2023, to April 30, 2023.

Sample size estimation and sampling

The sample size was based on a study by Muralidharan et al., which reported a 78% prevalence of MSD among dental practitioners in Andhra Pradesh, India [8]. Considering a non-response rate of 10% and an absolute precision of 5%, the required sample size was calculated to be 290. From the IDA's list of 789 registered dentists in Thrissur, participants were randomly selected using a simple random sampling technique.

Data collection

Dentists were approached to participate in the study through in-person interviews and Google Forms. Of the 290 participants, 30 agreed to in-person interviews, while the remaining 260 were sent Google Forms for data collection.

Study tools

The survey was divided into three sections; sociodemographic details, the standardized Nordic questionnaire, and the Work Ability Index (WAI) (see Appendices).

Sociodemographic details: Information collected included name, age, gender, specialization, years of practice, self-reported height and weight (for calculating BMI), average patients seen per day, nature of practice (full-time or part-time), hand dominance, marital status, family type, and place of residence.

Standardized Nordic questionnaire: The Nordic Musculoskeletal Questionnaire is a freely usable public instrument developed by a Nordic Council of Ministers project [9]. This tool assessed musculoskeletal discomfort in the past 12 months across various body areas (elbows, neck, shoulders, wrists/hands, upper back, lower back, hips/thighs, knees, and ankles/feet). Those reporting discomfort were also asked about their experiences in the past seven days.

Work Ability Index: WAI is a tool designed to evaluate an individual's ability to work, taking into account both physical and mental health factors. It includes questions about the nature of work, current work ability compared to one's highest ever, ability to meet job demands, current illnesses, and their impact on work performance. The WAI also assesses the individual’s mental capacity and predicts future work ability. The results yield a score ranging from 7 (indicating inability to work) to 49 (indicating full work ability). Based on this score, individuals are categorized into four groups: excellent (44-49), good (37-43), moderate (28-36), and poor (<27) [10]. 

Ethical considerations

The study protocol was reviewed and approved by the Institute Ethics Committee of Amrita School of Medicine, Kochi (Approval No.: ECASM-AIMS-2023-115). Participants interviewed in person provided written informed consent, with protocols in place to ensure privacy and confidentiality. For those participating via Google Forms, oral consent was obtained, and the form included a mandatory consent section that had to be completed before participation.

Statistical analysis

Categorical variables were summarized using frequencies and percentages. The prevalence of MSD was estimated by the proportion of participants reporting WMSD in at least one body region over the last year and the last week. Associations between sociodemographic variables and MSD in the knee or ankle over the last seven days were analyzed using the chi-square test. Unadjusted prevalence ratios (UPR) with 95% CIs were calculated, followed by log binomial regression analysis for variables with a p-value less than 0.2 in the unadjusted analysis to determine adjusted prevalence ratios (APR). Data analysis was conducted using STATA version 14 (StataCorp LLC, College Station, TX, USA).

## Results

The study population consisted of 290 dental professionals whose median age was 34 years. The sociodemographic characteristics and work ability index scores are provided in Table 1. 

**Table 1:** Sociodemographic details (N=290) BMI: Body Mass Index

Variable	N	%
Age (in years)		
25-35	167	57.6
36-45	63	21.7
46-55	42	14.5
56 and above	18	6.2
Gender		
Male	138	47.6
Female	152	52.4
Place of residence		
Urban	175	60.3
Rural	115	39.7
Marital status		
Married	249	85.9
Never married	36	12.4
Separate/widower	5	1.7
Family type		
Nuclear family	200	69
Joint family	90	31
Educational qualification		
Master of Dental Surgery (MDS)	91	31.4
Bachelor of Dental Surgery (BDS)	199	68.6
Work duration		
0-10 years	109	37.6
11-20 years	103	35.5
21-30 years	37	12.8
31-50 years	41	14.1
BMI (kg/m2)		
<18.5	6	2.1
18.5-24.9	123	42.4
25-29.9	133	45.9
≥30	28	9.7
Average patients seen per day		
0-5	66	22.8
6−10	95	32.8
11−20	85	29.3
20 above	44	15.2
Nature of practice		
Full time	212	73.1
Part-time	78	26.9
Hand dominance		
Right hand	283	97.6
Left hand	7	2.4
Work Ability Index score		
Excellent (44-49)	71	24.5
Good (37-43)	150	51.7
Moderate (28-36)	60	20.7
Poor (7−27)	9	3.1

The prevalence of MSD within the past 12 months was 86.9% (95% CI: 83.1 - 90.9) and in the past seven days was 42.4% (95% CI: 36.7 - 48.08). The region mostly affected by MSD for 12 months was the neck region accounting for 57.6% and the least affected region was the thigh region accounting for 9.3%. The region mostly affected by MSD in seven days was the lower back accounting for 21.4% and the least affected region was the elbow region accounting for 4.1%. The majority of the participants considered their work ability to be good (51.7%).

No significant association was obtained on doing bivariate analysis for MSD and sociodemographic factors for the period of 12 months and seven days. However, the variable age (p=0.037) and BMI (p=0.033) were found to be associated with MSD in the knee region in bivariate analysis (Table 2) and work duration (0.023) which was found to be associated with MSD in the ankle region (Table 3).

**Table 2:** Results of both bivariate and binary logistic regression analysis of MSD on knee region (N=290) MSD: musculoskeletal disorders, BMI: Body Mass Index, CI: confidence interval, MDS: Master of Dental Surgery, BDS: Bachelor of Dental Surgery, Ref: reference category

Variable	Total	MSD on knee (last 7 days)		Unadjusted odds ratio (95% CI)	Adjusted odds ratio (95% CI)	P-value
		Yes	No			
		n (%)	n (%)			
Age in years						
>34	136	15 (11.0)	121 (89.0)	2.60 (1.03-6.59)	2.20 (0.85-5.67)	0.103
≤34	154	7 (4.5)	147 (95.5)	Ref	Ref	−
Gender						
Female	152	12 (7.9)	140 (92.1)	1.10 (0.46-2.63)	−	−
Male	138	10 (7.2)	128 (92.8)	Ref	−	−
Place of residence						
Rural	115	10 (8.7)	105 (91.3)	1.29 (0.54-3.10)	−	−
Urban	175	12 (6.9)	163 (93.1)	Ref	−	−
Marital status						
Others	41	4 (9.8)	37 (90.2)	1.39 (0.45-4.33)	−	−
Married	249	18 (7.2)	231 (92.8)	Ref	−	−
Family type						
Joint family	90	8 (8.9)	82 (91.1)	1.30 (0.52-3.21)	−	−
Nuclear family	200	14 (7.0)	186 (93.0)	Ref	−	−
Educational qualification						
MDS	91	7 (7.7)	84 (92.3)	1.02 (0.40-2.60)	−	−
BDS	199	15 (7.5)	184 (92.5)	Ref	−	−
Work duration						
>8 years	138	13 (9.4)	125 (90.6)	1.65 (0.68-4.0)	−	−
<8 years	152	9 (5.9)	143 (94.1)	Ref	−	−
BMI						
≥25	161	17 (10.6)	144 (89.4)	2.93 (1.10-8.17)	2.50 (0.88-7.09)	0.086
<25	129	5 (3.9)	124 (96.1)	Ref	Ref	−
Average patients seen per day						
>10	129	12 (9.3)	117 (90.7)	1.55 (0.65-3.71)	−	−
≤10	161	10 (6.2)	151 (93.8)	Ref	−	−
Nature of practice						
Part-time	78	6 (7.7)	72 (92.3)	1.02 (0.39-2.71)	−	−
Full time	212	16 (7.5)	196 (92.5)	Ref	−	−
Hand dominance						
Left hand	7	1 (14.3)	6 (85.7)	2.08 (0.24-18.09)	−	−
Right hand	283	21 (7.4)	262 (92.6)	Ref	−	−

**Table 3:** Results of both bivariate and binary logistic regression analysis of MSD on ankle region (N=290) MSD: musculoskeletal disorders, BMI: Body Mass Index, CI: confidence interval, MDS: Master of Dental Surgery, BDS: Bachelor of Dental Surgery, Ref: reference category

Variable	Total	MSD on ankle (last 7 days)		Unadjusted odds ratio (95% CI)	P-value	Adjusted odds ratio (95% CI)	P-value
		Yes n (%)	No n (%)				
Age in years							
≤34	154	14 (9.1)	140 (90.9)	1.84 (0.72-4.71)	0.196	1.99 (0.46-8.53)	0.359
>34	136	7 (5.1)	129 (94.9)	Ref	−	Ref	−
Gender							
Female	152	15 (9.9)	137 (90.1)	2.41 (0.91-6.40)	0.07	2.77 (0.94-8.21)	0.066
Male	138	6 (4.3)	132 (95.7)	Ref	−	Ref	−
Place of residence							
Urban	175	13 (7.4)	162 (92.6)	1.07 (0.43-2.68)	0.879	−	−
Rural	115	8 (7.0)	107 (93.0)	Ref	−	−	−
Marital status							
Others	41	5 (12.2)	36 (87.8)	2.02 (0.70-5.86)	0.194	2.13 (0.64-7.14)	0.22
Married	249	16 (6.4)	233 (93.6)	Ref	−	Ref	−
Family type							
Nuclear family	200	17 (8.5)	183 (91.5)	1.10 (0.65-6.11)	0.218	−	−
Joint family	90	4 (4.4)	86 (95.6)	Ref	−	−	−
Educational qualification							
BDS	199	16 (8.0)	183 (92.0)	1.50 (0.53-4.24)	0.438	−	−
MDS	91	5 (5.5)	86 (94.5)	Ref	−	−	−
Work duration							
<8 years	152	16 (10.5)	136 (89.5)	3.13 (1.12-8.79)	0.023	4.23 (0.92-19.57)	0.065
>8 years	138	5 (3.6)	133 (96.4)	Ref	−	Ref	−
BMI							
≥25	161	13 (8.1)	148 (91.9)	1.33 (0.53-3.31)	0.541	1.99 (0.72-5.46)	0.184
<25	129	8 (6.2)	121 (93.8)	Ref	−	Ref	−
Average patients seen per day							
>10	129	10 (7.8)	119 (92.2)	1.15 (0.47-2.79)	0.764	−	−
≤10	161	11 (6.8)	150 (93.2)	Ref	−	−	−
Nature of practice							
Full time	212	16 (7.5)	196 (92.5)	1.19 (0.42-3.37)	0.74	−	−
Part-time	78	5 (6.4)	73 (93.6)	Ref	−	−	−
Hand dominance							
Left hand	7	2 (28.6)	5 (71.4)	5.56 (1.01-30.57)	0.084	7.30 (1.11-48.21)	0.039
Right hand	283	19 (6.7)	264 (93.3)	Ref	−	Ref	−

## Discussion

In this study, we examined the prevalence of MSD, work ability, and the factors associated with MSD among dentists in Thrissur. The findings revealed that the prevalence of MSD among dentists was 86.9% within the past 12 months and 42.1% within the past seven days.

The rates in our study indicate a high prevalence of MSD in this population, consistent with a few previous studies. Some of the studies with a high prevalence of MSD within the past 12 months include a study conducted in the Czech Republic among dentists, which reported a prevalence rate of 96.9% [11]. Furthermore, a study conducted among dentists and dental hygienists in Karachi reported a prevalence of 96.7% [2]. A study in Karachi by Ali et al. reported a prevalence of 88.8%, which was almost similar to the findings of the current study [12]. In contrast, a study conducted in Indonesia reported a lower prevalence rate of 63.5% [13]. Another study conducted among dentists in Davangere, Karnataka reported a prevalence rate of 34.71% for MSD [14]. The lower prevalence rate in this study could be attributed to the smaller sample size, which consisted of 97 participants [14].

The present study revealed that the neck region was the most frequently affected area by MSD, with 57.6% of participants reporting issues within a 12-month period. This observation is consistent with similar studies conducted among dentists in China, where the neck region was also reported as the most affected area, accounting for 83.8% [15].

In a study conducted in Jeddah, Saudi Arabia, the neck region was identified as the most affected area among both males (79.5%) and females (90.7%) [16]. A study conducted among dentists in Rajahmundry, India, showed the highest prevalence of MSD in the neck region, with a rate of 24% [17]. Additionally, a study conducted in Karachi identified the highest prevalence of shoulder issues among participants, accounting for 36.24% [12]. It was observed that the lower back region was the most commonly reported site of MSD for seven days (21.4%).

In the present study, 24.5% of participants reported excellent work ability, 51.7% reported their work ability as good, 20.7% as moderate, and 3.1% as poor. This finding suggests that a majority of the participants continue to work despite experiencing musculoskeletal conditions. In a study conducted in Sweden among dentists, the Work Ability Index (WAI) was assessed, revealing that 0.6% of participants had poor work ability, while 5.8% had moderate, 34.9% had good, and 58.7% had excellent work ability [18].

The study revealed a significant association between MSD in the knee region for a seven-day duration and two sociodemographic variables: age and BMI. Participants aged 34 years and above had 2.60 times higher odds of developing MSD compared to those below the age of 34. Similarly, individuals with a BMI of 25 and above had a 2.9 times greater risk of developing MSD than those with a BMI below 25. Also, a significant association was found between MSD in the ankle region for a seven-day duration and work duration. Participants with a work duration of ≤8 years were found to have a three times higher risk of developing MSD compared to those with >8 years of work duration. However, these findings may be influenced by the limited sample size employed in the study. Furthermore, it is noteworthy to mention that although certain associations were found to be significant in the initial analysis, they did not remain significant in the regression analysis.

In this study, no significant association was found between the prevalence of MSD and sociodemographic variables for both within the last 12 months and seven days. Additionally, no significant association was found between work ability and the prevalence of MSD, both for the 12-month period and the seven-day duration.

Among the studies examining MSD in various professional groups, one conducted in Egypt by Hassan et al. reported a prevalence of 88.4% among dentists and 58.5% among physicians [19]. These findings suggest that dentists face a higher risk of MSD compared to physicians. Another research study carried out in Chennai involved the examination of 140 healthcare professionals, comprising dentists, laboratory technicians, nurses, physicians, and physiotherapists from different clinical departments [20]. It revealed that nurses had the highest incidence of WMSD at 55.5%, followed by physiotherapists (55%), dentists (53.5%), lab technicians (38.7%), and physicians (38%) [20].

The current study addresses a significant research gap by exploring MSD among dentists. However, a potential limitation of this study is its reliance on self-reported data, which carries an inherent risk of overestimation or underestimation of MSD. Another limitation of this study is that we did not include specialists. The prevalence of MSD may differ among dental sub-specialties. For instance, in lingual orthodontics, where practitioners often maintain a bending posture while working on the palatal side, lower back pain may be more prevalent [21]. The cross-sectional nature of the study restricts the ability to establish causality between observed factors and MSD. Additionally, since the study was conducted in only one district, its findings may have limited generalizability to other regions or the broader population of dentists in India.

## Conclusions

The prevalence of WMSD among dentists is notably high. Addressing ergonomic factors, enhancing awareness and education, and implementing effective organizational policies can significantly reduce the burden of MSD. Such initiatives would improve dentists' work ability and enhance their overall well-being and the quality of their professional practice.

## Appendices

**Table 4:** Questionnaire used in the study to assess demographics, occupational health, and musculoskeletal symptoms

Section	Question	Options
Section 1	1. Name	________
	2. Age	________
	3. Gender	a. Male
		b. Female
		c. Others
	4. Place of residence	i. Urban
		ii. Rural
	5. Marital status	i. Married
		ii. Never married
		iii. Widower
		iv. Separated
	6. Family type	i. Nuclear Family
		ii. Joint Family
	7. Qualification	a. MDS
		b. BDS
	8. Work duration	________
	9. Height	________
	10. Weight	________
	11. BMI	________
	12. Average patients seen per day	________
	13. Nature of practice	a. Full-time
		b. Part-time
	14. Hand used	a. Right-handed
		b. Left-handed
Section 2	Have you had trouble (ache, pain, discomfort) in the last 12 months in:	
	1. Neck	a. No
		b. Yes
	Have you been prevented from normal work because of the trouble in the last 12 months?	a. Yes
		b. No
	Have you had trouble in the last 7 days?	a. Yes
		b. No
	2. Shoulders	a. No
		b. Yes, right shoulder
		c. Yes, left shoulder
		d. Yes, both shoulders
	Have you been prevented from normal work because of the trouble in the last 12 months?	a. Yes
		b. No
	Have you had trouble in the last 7 days?	a. Yes
		b. No
	3. Elbows	a. No
		b. Yes, right elbow
		c. Yes, left elbow
		d. Yes, both elbows
	Have you been prevented from normal work because of the trouble in the last 12 months?	a. Yes
		b. No
	Have you had trouble in the last 7 days?	a. Yes
		b. No
	4. Wrists/hands	a. No
		b. Yes, right wrist/hand
		c. Yes, left wrist/hand
		d. Yes, both wrist/hands
	Have you been prevented from normal work because of the trouble in the last 12 months?	a. Yes
		b. No
	Have you had trouble in the last 7 days?	a. Yes
		b. No
	5. Upper back	a. No
		b. Yes
	Have you been prevented from normal work because of the trouble in the last 12 months?	a. Yes
		b. No
	Have you had trouble in the last 7 days?	a. Yes
		b. No
	6. Lower back	a. No
		b. Yes
	Have you been prevented from normal work because of the trouble in the last 12 months?	a. Yes
		b. No
	Have you had trouble in the last 7 days?	a. Yes
		b. No
	7. Hips/thighs	a. No
		b. Yes
	Have you been prevented from normal work because of the trouble in the last 12 months?	a. Yes
		b. No
	Have you had trouble in the last 7 days?	a. Yes
		b. No
	8. Knees	a. No
		b. Yes
	Have you been prevented from normal work because of the trouble in the last 12 months?	a. Yes
		b. No
	Have you had trouble in the last 7 days?	a. Yes
		b. No
	9. Ankles/feet	a. No
		b. Yes
	Have you been prevented from normal work because of the trouble in the last 12 months?	a. Yes
		b. No
	Have you had trouble in the last 7 days?	a. Yes
		b. No
Section 3	1. Work demand	a. Psychologically demanding
		b. Physically demanding
		c. Both physically and psychologically demanding
	2. Current work ability (0-10 scale)	________
	3. Work ability with respect to physical demands	a. Very good (5)
		b. Rather good (4)
		c. Moderate (3)
		d. Rather poor (2)
		e. Very poor (1)
	4. Work ability with respect to mental demands	a. Very good (5)
		b. Rather good (4)
		c. Moderate (3)
		d. Rather poor (2)
		e. Very poor (1)
	5. Current diseases (mark yes/no/physician diagnosed)	Injury due to accident
		Musculoskeletal disease
		Cardiovascular diseases
		Respiratory disease
		Mental disorder
		Neurological or sensory disease
		Digestive disease
		Genitourinary disease
		Skin disease
		Tumor or cancer
		Endocrine or metabolic disease
		Blood diseases
		Birth defects
		Other disorders
	6. Estimated work impairment due to diseases	a. No hindrance
		b. Able to do job but with symptoms
		c. Must sometimes slow down/change methods
		d. Must often slow down/change methods
		e. Able to do only part-time work
		f. Unable to work
	7. Illness within last year	a. None (5)
		b. Max. 9 days (4)
		c. 10-24 days (3)
		d. 25-99 days (2)
		e. 100-354 days (1)
	8. Estimation of work ability in 2 years	a. Unlikely (1)
		b. Not certain (4)
		c. Relatively certain (7)
	9. Mental capacities (last 3 months)	
	Have you been able to enjoy your regular activities?	a. Often (4)
		b. Rather often (3)
		c. Sometimes (2)
		d. Rather seldom (1)
		e. Never (0)
	Have you been active and alert?	a. Often (4)
		b. Rather often (3)
		c. Sometimes (2)
		d. Rather seldom (1)
		e. Never (0)
	Have you felt hopeful about the future?	a. Often (4)
		b. Rather often (3)
		c. Sometimes (2)
		d. Rather seldom (1)
		e. Never (0)
